# Supplementary material for: Evaporation-Rate Control of Water Droplets on Flexible Transparent Heater for Sensor Application
Source: Sensors (Basel). 2019 Nov 12;19(22):4918. doi: 10.3390/s19224918 (PMC6891349; doi:10.3390/s19224918)
Supplement: Supplementary file 1 [file sensors-19-04918-s001.pdf]

sensors

Supplementary material

Evaporation-rate Control of Water Droplets on Flexible Transparent Heater for Sensor Application

Jaesoung Park [1,†], Suhan Lee [2,†], Dong-Ik Kim [3], Young-You Kim [4] and Samsoo Kim [1], Han-Jung Kim [1,*] and Yoonkap Kim [1,*]

1 Convergence Materials Research Center, Gumi Electronics & Information Technology Research Institute (GERI), Gumi 39171, Korea
2 Convergence Medical Devices Research Center, Gumi Electronics & Information Technology Research Institute (GERI), Gumi 39253, Korea
3 Center for Integrated Smart Sensors (CISS), Korea Advanced Institute of Science and Technology (KAIST), Daejeon 34141, Korea
4 Department of Physics, Kongju National University, Gongju 32588, Korea
* Correspondence: hjkim0321@geri.re.kr (H-J.K.); yoonkap@geri.re.kr (Y.K.);
Tel.: +82-54-479-2133 (H-J.K.); +82-54-479-2120 (Y.K.)
† These authors contributed equally to this work.

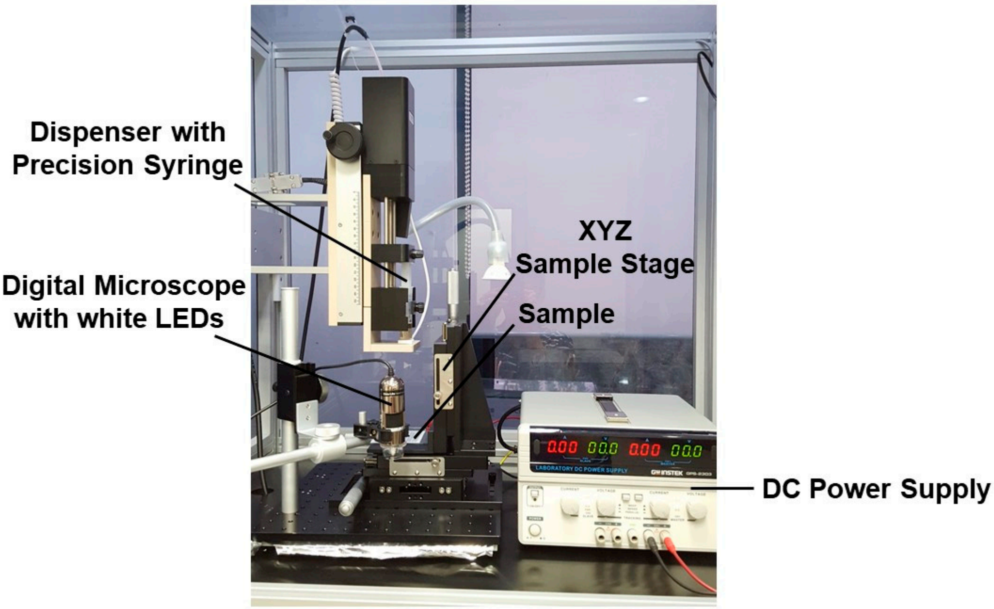

Figure S1. Real-time microscope imaging system for observing water droplet evaporation on the transparent heater surfaces.

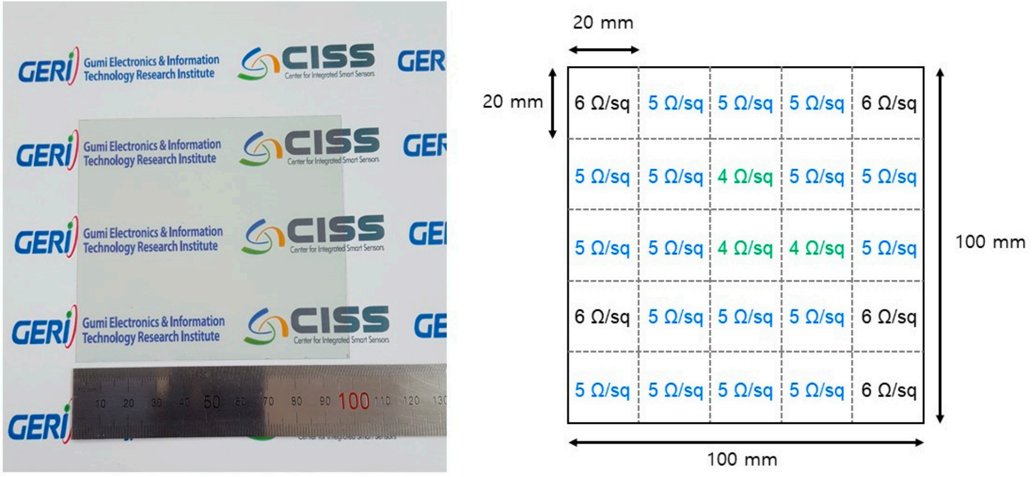

Figure S2. Photograph of the fabricated ITO/Ag/ITO multilayer transparent heater and uniformity of the measured sheet resistance (4–6 Ω/sq) of the heater.

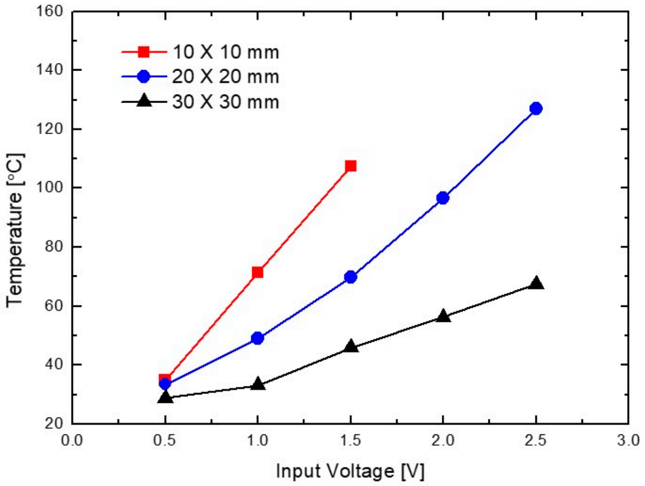

Figure S3. Steady-state temperature of ITO/Ag/ITO multilayer transparent heater according to input voltage and heater size.

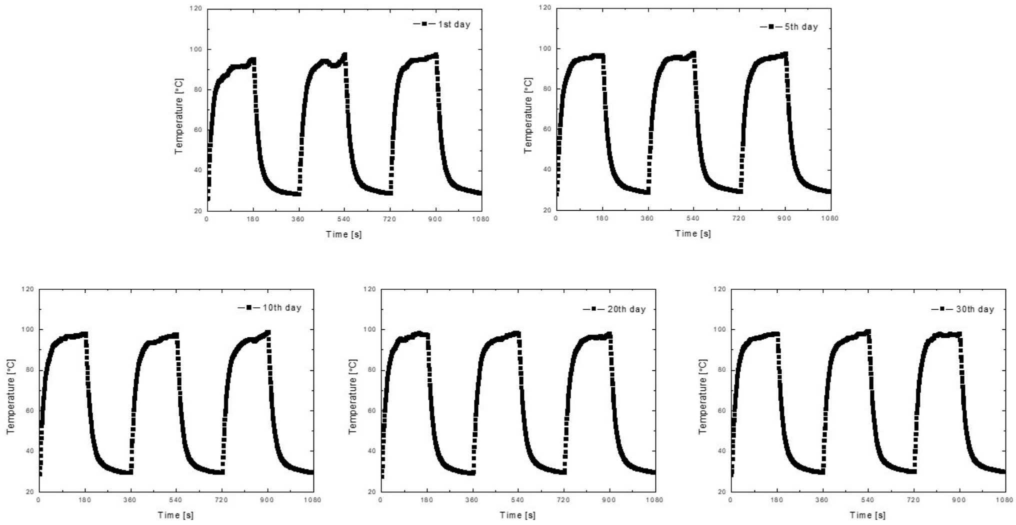

Figure S4. Reproducibility of heat-generation performance of the ITO/Ag/ITO multilayer transparent heater for 30 days.

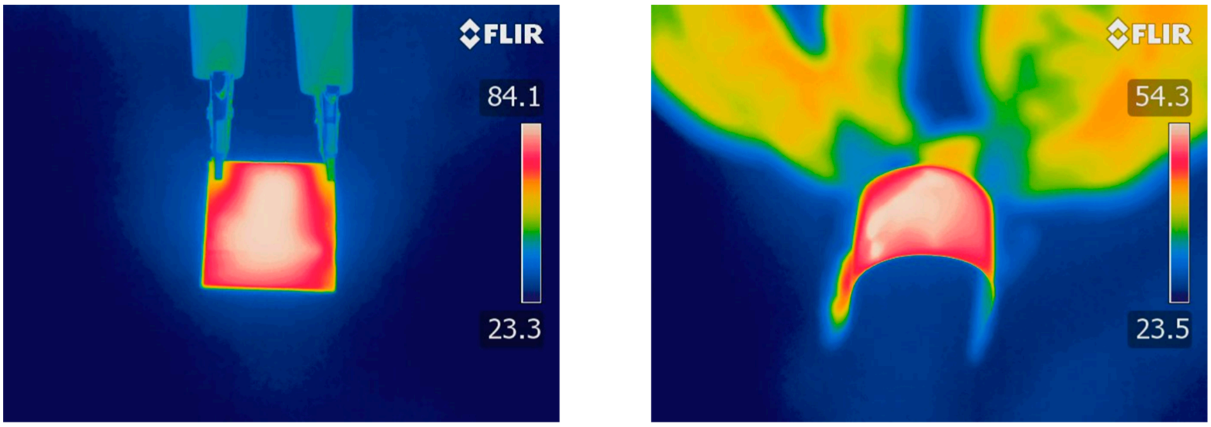

Figure S5. IR Images without any contacts underneath the heater.

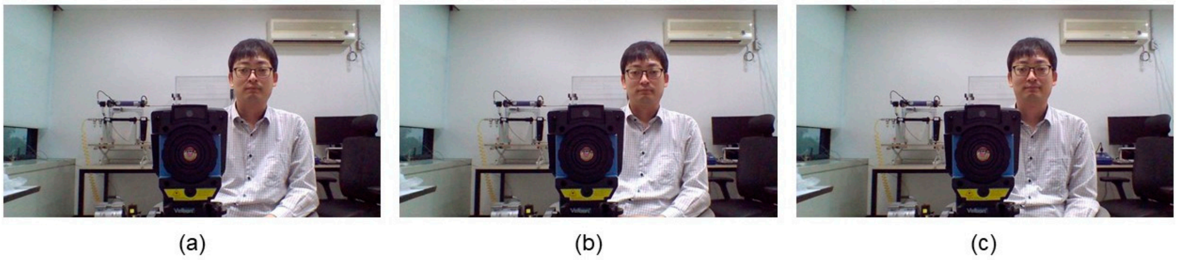

Figure S6. Images captured by the webcam (a) without and (b) with the transparent heater, and (c) during operation at 50 °C.

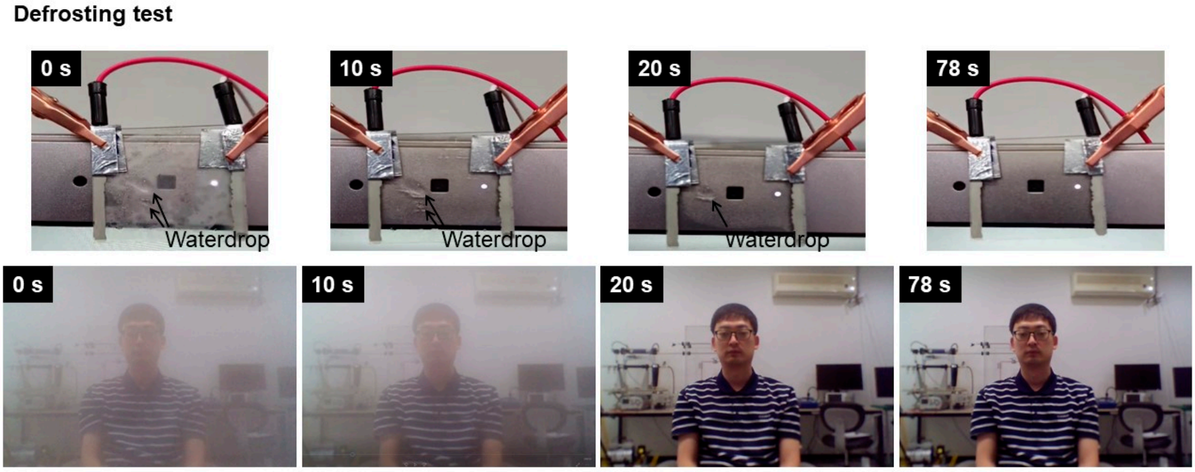

Figure S7. Defrosting test results using a transparent heater.

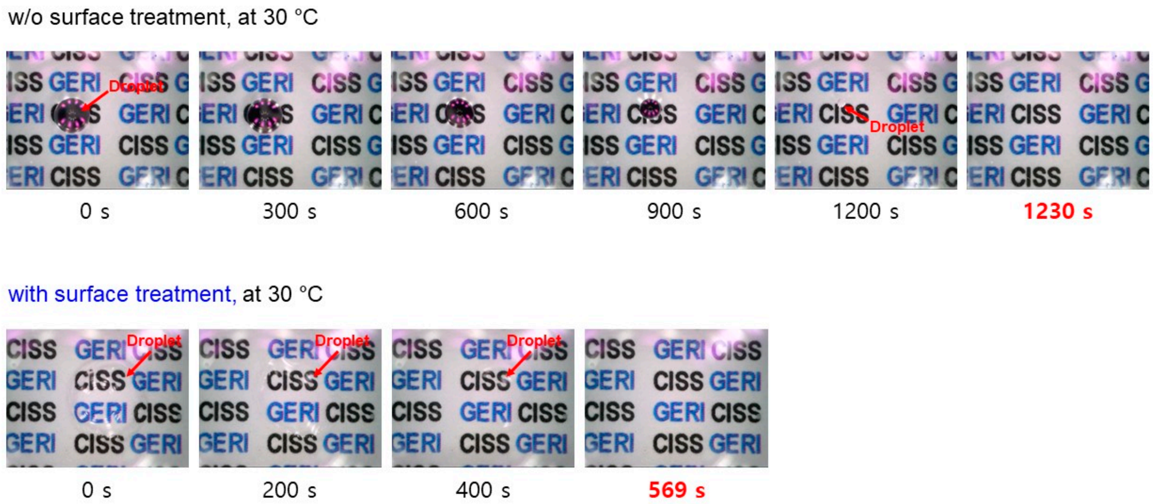

Figure S8. Water droplet evaporation on the surfaces of the ITO/Ag/ITO multilayer transparent heaters (heat-generation temperature of 30 °C) with different wetting properties.

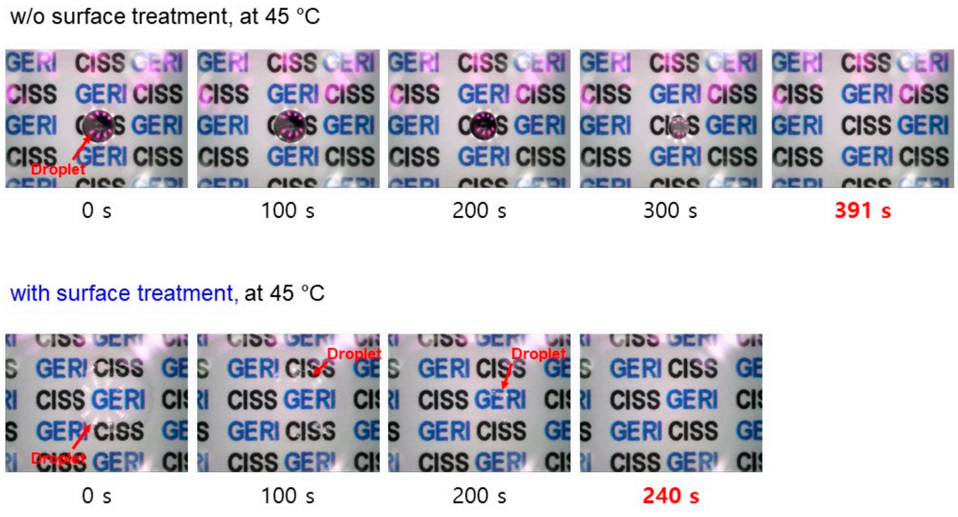

Figure S9. Water droplet evaporation on the surfaces of the ITO/Ag/ITO multilayer transparent heaters (heat-generation temperature of 45 °C) with different wetting properties.

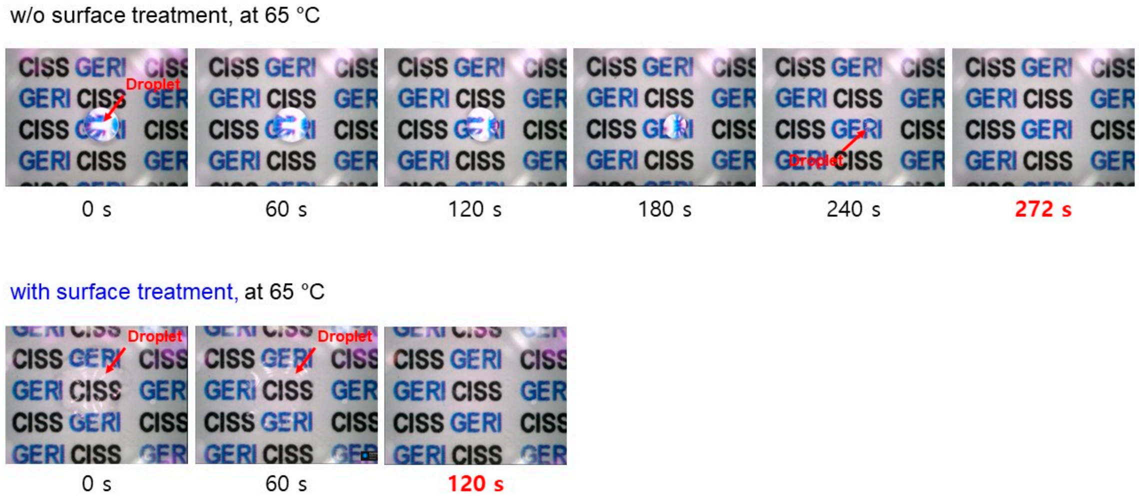

Figure S10. Water droplet evaporation on the surfaces of the ITO/Ag/ITO multilayer transparent heaters (heat-generation temperature of 65 °C) with different wetting properties.
